# A metacommunity ecology approach to understanding microbial community assembly in developing plant seeds

**DOI:** 10.3389/fmicb.2022.877519

**Published:** 2022-07-22

**Authors:** Gillian E. Bergmann, Johan H. J. Leveau

**Affiliations:** Department of Plant Pathology, University of California-Davis, Davis, CA, United States

**Keywords:** priority effects, selection, dispersal, metacommunities, epiphytes, endophytes, community assembly, seed microbiota

## Abstract

Microorganisms have the potential to affect plant seed germination and seedling fitness, ultimately impacting plant health and community dynamics. Because seed-associated microbiota are highly variable across individual plants, plant species, and environments, it is challenging to identify the dominant processes that underlie the assembly, composition, and influence of these communities. We propose here that metacommunity ecology provides a conceptually useful framework for studying the microbiota of developing seeds, by the application of metacommunity principles of filtering, species interactions, and dispersal at multiple scales. Many studies in seed microbial ecology already describe individual assembly processes in a pattern-based manner, such as correlating seed microbiome composition with genotype or tracking diversity metrics across treatments in dispersal limitation experiments. But we see a lot of opportunities to examine understudied aspects of seed microbiology, including trait-based research on mechanisms of filtering and dispersal at the micro-scale, the use of pollination exclusion experiments in macro-scale seed studies, and an in-depth evaluation of how these processes interact *via* priority effect experiments and joint species distribution modeling.

## Introduction

The plant microbiota, defined here as the community of bacteria, fungi, archaea, viruses, and other microscopic organisms that live on (i.e., epiphytically) or in (i.e., endophytically) plant tissues (Hardoim et al., 2015), confer many services as well as disservices to their hosts, including disease development and defense (Busby et al., 2016), protection against herbivory (Shikano et al., 2017), tolerance of abiotic stress (Rodriguez et al., 2004), and aid in nutrient uptake (Christian et al., 2019). These microbial communities associate with all plant tissues (Hardoim et al., 2015), including seeds (Mundt and Hinkle, 1976; Ganley and Newcombe, 2006; U’Ren et al., 2009; Hodgson et al., 2014; Barret et al., 2015). Seeds play a major role in plant communities as agents of dispersal, genetic diversity, and regeneration (Fenner and Thompson, 2005), and they have significant economic and social value through agriculture (Nabhan, 2012). Seeds also are a major bottleneck in natural plant populations, as they face heightened mortality from abiotic stressors, pests, pathogens, and predators (Bever et al., 2015). As the initial source of inoculum in a plant’s life cycle, seed microbes are can be transmitted across plant generations and have lifelong impacts (Barret et al., 2015; Abdelfattah et al., 2021). Consequently, understanding how seeds acquire and interact with their microbiota, for example, *via* priority effects (Fukami, 2015; Ridout et al., 2019; Johnston-Monje et al., 2021) or according to the Primary Symbiont Hypothesis (Newcombe et al., 2018), has implications for improving seed health, seedling establishment, and plant community structure. Previous work on seed microbiota has primarily taken a pattern-based approach to studying assembly processes (e.g., Rezki et al., 2018). Such an approach uses culturing (Ganley and Newcombe, 2006; U’Ren et al., 2009; Heitmann et al., 2021) and/or next-generation sequencing (e.g., Barret et al., 2015; Rezki et al., 2018; Prado et al., 2020; Bergmann and Busby, 2021; Fort et al., 2021) to compare, contrast, and correlate patterns in microbial community composition, diversity, and species co-occurrences. Typically, however, these community data provide limited (i.e., mostly indirect) insights into processes such as dispersal, microbe-plant interactions, and microbe-microbe interactions. Given that seed microbial communities are highly variable across individual plants, plant species, and locations (Simonin et al., 2021), such pattern-based data cannot always be used to predict assembly outcomes. Moreover, such studies often consider how these assembly processes occur at a single spatial scale (e.g., between sites or plant populations; Klaedtke et al., 2016; Adam et al., 2018; Chartrel et al., 2021). We hypothesize that a mechanistic, multi-scale approach would provide a more complete understanding of how microbial communities assemble in seeds, with the field of metacommunity ecology providing a theoretical framework for such an approach.

**Figure 1:**
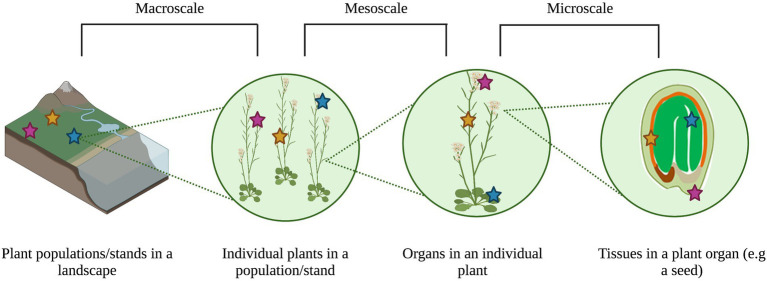
Variation in plant microbial communities can be observed at the macro-scale (i.e., meters to kilometers, or within and among sampling sites), meso-scale (i.e., centimeters to meters, or within and among plant organs to individuals), and micro-scale (i.e., nanometers to centimeters, or within and among plant organs) scales. The different colored stars represented distinct microbial communities at each scale. Image created through BioRender.com.

Metacommunity theory accounts for the interaction between ecological processes and habitat heterogeneity across spatio-temporal scales to impact community patterns (Leibold and Chase, 2018). This emphasis on multiple scales and heterogeneity can help explain the main drivers of community assembly and patterns of biodiversity and co-occurrence (Leibold and Chase, 2018). Plant-associated microbial communities vary widely across environmental gradients (Barge et al., 2019) and host genetics (Wagner et al., 2016) from the levels of tissues (Cregger et al., 2018) to populations (Bergmann and Busby, 2021). As such, treating individual plants as heterogeneous habitats for microorganisms that are embedded in a larger, heterogeneous landscape of multiple plants representing different species provides a new approach to observing, testing, and modeling drivers of microbial community variation (Figure 1). However, the study of microbiota through a metacommunity lens is still relatively new, both for animals (Miller et al., 2018) and plants (Christian et al., 2015; Borer et al., 2016), and the plant seed represents a relatively understudied microbiome in this context.

In this review, we address how mechanisms of seed microbial community assembly have been studied at different spatial micro-, meso-, and macro-scales (Figure 1), and advocate for a metacommunity-based approach to seed microbiology in future work. For this review, we use the definition of community assembly from Fukami (2010): “the construction and maintenance of local communities through sequential, repeated immigration of species from the regional species pool.” Additionally, most studies that we cover in our review will be focused on fungi and bacteria (Table 1). We acknowledge that archaea, viruses, and protists are frequent members of plant-associated microbial communities (Trivedi et al., 2020), many plant viruses are seed-transmitted (Sastry, 2013), and viruses can play a major role in the diversity and function of soil microbial communities (Albright et al., 2022). However, the ecological roles of these microbes in plant microbial communities, including those of seeds, are still largely unknown. As such, we cannot speak on their contributions to seed microbiota assembly here and recommend new research on these microbes in seeds. We will first summarize the modes of microbial acquisition into seeds, and how metacommunity ecology frames this assembly process. We then discuss studies of seed microbiome assembly which examine the processes of filtering, species interactions, dispersal, and ecological drift. We specifically highlight studies that address assembly processes during seed development and maturation, as these stages are understudied compared to seed dormancy and germination, and they are likely the source of microbes that persist between plant generations (Chesneau et al., 2020). Lastly, we suggest future lines of research to gain a more mechanistic, scale-explicit understanding of seed microbiome assembly.

**Table 1:** Commonly found fungal and bacterial taxa in seeds across broad plant groups.

Plant group	Commonly observed^a^ fungal taxa	Commonly observed^a^ bacterial taxa	Fungal taxa with known phytopathogenic representatives^b^	Bacterial taxa with known phytopathogenic representatives^b^
Angiosperms (monocots; e.g., grasses)	*Alternaria, Aspergillus, Colletotrichum, Epichloë, Fusarium*	*Pantoea, Pseudomonas, Sphingomonas, Burkholderia, Enterobacter*	*Alternaria, Aspergillus, Colletotrichum, Fusarium*	*Pseudomonas*
Angiosperms (dicots; e.g., brassicas, the rose family)	*Alternaria, Cladosporium, Cryptococcus, Stemphylium, Aureobasidium*	*Pseudomonas, Pantoea, Bacillus, Acinetobacter, Erwinia*	*Alternaria, Cladosporium, Stemphylium*	*Pseudomonas, Erwinia*
Gymnosperms (e.g., pines, firs, cypresses)	*Cladosporium, Hormonema, Trichoderma, Alternaria, Caloscypha*	*Pseudomonas, Bacillus, Enterobacter, Erwinia, Lysinibacillus*	*Alternaria, Cladosporium, Hormonema*	*Pseudomonas, Erwinia*

a Commonly observed taxa are defined here as the top five most mentioned among the most abundant genera that were identified in a selection of representative papers (Supplementary Table 1). This table is not meant to be exhaustive or quantitative and only offers a qualitative sense for the types of fungal and bacterial taxa that these studies recognized as most likely to find associated with seeds. For more extensive reviews of bacteria and fungi found across plants, we recommend referring to Simonin et al. (2021) and Newcombe et al. (2022). ^b^Putative pathogens were defined as any of the commonly observed genera that have pathogenic strains reported in the UC Integrated Pest Management Plant Disease List (http://ipm.ucanr.edu/PMG/menu.disease.html).

## Acquisition modes of seed microbiota

Plant seeds are generally composed of three tissues: a seed coat which provides physical protection (Belmonte et al., 2013), an embryo which is the precursor to the seedling and is made up of an immature root, a stem, and one or more embryonic leaves (Boesewinkel and Bouman, 1995; Bewley et al., 2012; Bewley and Nonogaki, 2017), and an endosperm which typically consists of carbohydrates and proteins and provides nutrition for the embryo during germination and growth before photosynthesis can occur (Berger, 1999). Seed development involves three stages (Belmonte et al., 2013; Bewley and Nonogaki, 2017). Following fertilization by pollen, the egg cells divide and differentiate into the embryo and endosperm tissues, in a process called histodifferentiation (Bewley and Nonogaki, 2017). Next, the cells expand and mature with reduced division, and seed mass increases during this filling stage, as nutrient reserves are deposited into the endosperm (Bewley and Nonogaki, 2017). After this, nutrient accumulation declines, and the seed goes into maturation drying and loses about 10%–15% moisture content before it is ready to be dispersed (Angelovici et al., 2010; Bewley and Nonogaki, 2017).

During seed development, microbes may enter the seed tissues *via* three distinct routes of transmission: vertical, floral, and horizontal (Maude, 1996). Vertical transmission involves microbes traveling from other organs (e.g., stems, roots) of the mother plant to the developing embryo. Such transmission is cited as an ecologically important way for plants to inherit beneficial microbes across generations (Barret et al., 2015) and for seed-associated pathogens to disperse (Darsonval et al., 2008; Darrasse et al., 2010, 2018; Barret et al., 2016). Vertical transmission has long been observed in grasses, which are hosts to clavicipitaceous fungal endophytes (i.e., fungi in the family Clavicipitaceae; Porras-Alfaro and Bayman, 2011) such as *Epichloe* (Schardl et al., 2004; Truyens et al., 2015). Vertical seed transmission has also been observed for non-clavicipitaceous endophytes in *Setaria viridis* (Rodríguez et al., 2020), *Triticum* (Vujanovic et al., 2019), *Quercus* (Fort et al., 2021), and other plants (Newcombe et al., 2018). Floral transmission of microbes into seeds has been studied extensively for pathogens such as *Monilinia vaccinii-corymbosi* in blueberry (Ngugi and Scherm, 2004) and *Acidovorax citrulli* in watermelon (Walcott et al., 2003; Lessl et al., 2007; Dutta et al., 2012, 2015). However, flower-to-seed transmission has also been observed for commensal and beneficial bacteria, for example in *Brassica napus* (Prado et al., 2020). The microbial contributions of the vertical and floral transmission pathways are likely to vary based on a plant species’ pollination mode (Chesneau et al., 2020). Horizontal transmission is the acquisition of seed microbes from the environment, either prior to or after the maturation of the seed as it is still attached to the mother plant (Deckert et al., 2019) or as matured seed disperse and becomes colonized from sources such as air (suggested in Gandolfi et al., 2013), water (Crocker et al., 2016), animals (Correia et al., 2019; Lash et al., 2020), soil (Fort et al., 2021), and other seeds in storage (suggested in Bergmann and Busby, 2021). Seed dormancy and germination are likely to represent a very active period of such horizontal transmission, as soil microbes interact with seed exudates and pre-existing microorganisms on and within the seed (Nelson, 1990; Ofek et al., 2011).

## The case for studying seed microbiome assembly through a metacommunity lens

The metacommunity concept was formally described by Leibold et al. (2004), who defined metacommunities as sets of local communities that are interconnected by dispersal. This definition arose out of a need to better account for spatio-temporal scales in ecological studies (Leibold et al., 2004), and also included the impacts of dispersal and habitat heterogeneity on community patterns (Leibold and Chase, 2018). Since it was first described, metacommunity theory has adopted Vellend (2010, 2016) synthesis that community assembly and composition are driven by four categories of processes: (1) abiotic and host filtering, (2) species interactions, (3) dispersal, and (4) ecological drift (Leibold and Chase, 2018). Categories 1 and 2 (filters and interactions) represent a deterministic or niche-based process of selection where differences in fitness between taxa, species, or guilds lead to differences in their abundances (Vellend, 2010). Dispersal is the stochastic (or neutral, chance-based) process by which taxa move between local communities (Vellend, 2010). Finally, drift is the stochastic fluctuation in species abundances, often due to chance birth, death, and migration events (i.e., demographic stochasticity; Leibold and Chase, 2018).

Framing plant microbiomes as metacommunities provides an integrated view of the drivers of their composition, function, and evolution, and of the impacts of these drivers on host health (Mihaljevic, 2012). Traditional metacommunity ecology states that filtering and species interactions occur at the local scale (i.e., less than one square meter to several square kilometers; Cornell and Lawton, 1992), while dispersal and drift occur at the regional scale (i.e., many square kilometers; Cornell and Lawton, 1992; Leibold and Chase, 2018). However, categorizing processes as “local” or “regional” is relative to the community that is being studied, and depends on the scales of interest and on defining the boundaries between a local community and a regional metacommunity. For plant microbiota, including those associated with seeds, the terms “local” and “regional” are contextual because microbes primarily behave at very small scales (i.e., the micrometer scale; Leveau et al., 2018; Leveau, 2019), although they can be affected by much larger scale factors (e.g., plant genotype or climate gradients across kilometers; Dini-Andreote et al., 2020). Furthermore, microbes can be ubiquitous across habitats at multiple scales, blurring the boundaries between patches of local communities in the landscape of interest (Mony et al., 2020). As we apply the first principles of metacommunity ecology to plant and seed microbiology below, we will therefore use three categories of spatial scale: macro- (i.e., meters to kilometers, or within and among sampling sites), meso- (i.e., centimeters to meters, or within and among plant organs to individuals), and micro- (i.e., nano-to centimeters, or within and among plant organs) scales. Integrating the study of assembly processes across these three scales should give a more complete picture of how microbial communities are assembled, and how emergent community patterns occur at individual scales (Ricklefs, 1987; Shade et al., 2018).

## Deterministic processes: Abiotic filtering, host filtering, and species interactions

### Abiotic filtering

Several studies have shown that seed microbial communities differ significantly across geographic locations, i.e., at the macro-scale, for example in *B. napus* (Morales Moreira et al., 2021)*, Elymus nutans* (Guo et al., 2020)*, Phelipanche ramosa* (Huet et al., 2020) and *Pseudotsuga menziesii* (Bergmann and Busby, 2021). For most of these studies, the abiotic factors that are important for structuring these seed microbial communities remain to be identified. However, we can assume that these factors are similar to the ones that drive macro-scale differences in the microbial communities on/in other parts of the plant. In communities associated with leaves, roots, and fruits, such factors include temperature (Zimmerman and Vitousek, 2012), precipitation (Zimmerman and Vitousek, 2012; Barge et al., 2019), humidity (Bokulich et al., 2014), and soil conditions (e.g., available cations, soil pH; Johnston-Monje et al., 2016). In a study of aboveground microbial communities in *Vitis vinifera,* Bokulich et al. (2014) found that fungal communities of seeded fruit were associated with net precipitation, relative humidity, and temperature. During dormancy in the soil, the bacterial communities of *Noccaea caerulescens* seeds were correlated with soil pH and cation composition (Durand et al., 2020).

Not much is known either about variation in seed microbial community as a function of abiotic factors at the meso-and micro-scales, although again, much can be learned from studies on other aboveground plant tissues. At the meso-scale of an individual plant, microbial communities can vary with tissue location such as canopy height in trees. Unterseher et al. (2007) cultured fungi from leaves at different canopy heights in several tree species. They found that species richness was greater in the lower canopy. Harrison et al. (2016) went on to use next-generation sequencing in a survey of the needle fungi of *Sequoia sempervirens* at different height positions, and found that there were distinct communities present at each height across trees. While they did not measure microclimate variables within the trees sampled, they suggested that the observed variation could be attributed to the amount of sunlight (Harrison et al., 2016). At the micro-scale (i.e., across parts of a single plant organ), factors such as exposure to ultraviolet (UV) radiation and water availability can also be important. Hayes et al. (2021) described variation in the bacterial communities and UV radiation along individual flower petals in two sunflower species. They found that while there was no significant difference in community composition along petals, there was variation in UV tolerance in association with source petal position (Hayes et al., 2021). Another potentially important factor may be water availability, which has been shown to affect bacterial survival, growth, and movement on leaf surfaces (Doan et al., 2020).

For many macro-scale studies, a major limitation is the use of location as a proxy for environmental conditions, which precludes linking variation in microbial communities to specific environmental factors. Because site effects are impacted by environmental, spatial, and temporal factors, it can be difficult to parse out how location and environment influence seed microbiota (Bergmann and Busby, 2021). Also, most of these studies do not explore if and how environmental conditions actually select for microbial traits and taxa. *In vitro* experiments suggest that there is potential for environmental filtering, as demonstrated by thermotolerance in fungal endophytes of desert plants (Sangamesh et al., 2018), salt stress tolerance in fungal root endophytes (Gaber et al., 2020), water stress tolerance in bacterial endophytes (Yandigeri et al., 2012; Daranas et al., 2018), and oxidative stress tolerance in the fungal endophyte *Epichloë festucae* (Eaton et al., 2008). Similar characterization of seed microbial tolerance and survival when challenged with different environmental conditions could provide a more mechanistic understanding of abiotic filtering. Such studies would be particularly insightful at the micro-and meso-scales.

### Host filtering

Variation in plant microbial communities is often studied and interpreted as a result of plant genetics, which represents filtering through host selection. Studies at the macro-and meso-scales have revealed that plant genetics can significantly impact microbial community composition in different parts of the plant, although seeds are clearly underrepresented in the body of literature on this topic. Microbial community variation has been associated with specific genes in leaves and roots of various plants (Horton et al., 2014; Wagner et al., 2016; Deng et al., 2021), an approach that has not yet been applied to seeds, as far as we know. Seed line (i.e., familial line of descent traced to an individual seed) has been weakly associated with microbial community variation in *Zea mays* (Yang et al., 2020) and *B. napus* (Morales Moreira et al., 2021). Seed accessions (i.e., populations) of *Oryza* were also associated with variation in bacterial and fungal community composition, with significant compositional shifts between wild and domesticated accessions (Kim et al., 2020). In a study of the bacterial and fungal communities associated with grapes, Singh et al. (2018) found that host genotype had an impact particularly within individual sites, whereas abiotic conditions better explained microbial community variation between sites. This is consistent with the notion that host effects are difficult to reveal without carefully controlling for environmental factors, which would suggest, by extension, that environmental factors may have a greater relative impact on seed microbiota than plant genotype. A recent study showed however that the fungal community composition of *Quercus petraea* internal seed tissue was largely influenced by the mother plant, with only weak significant environmental influences (Fort et al., 2021).

Studying the roles of plant functional traits in seed microbiome assembly and dynamics provides the mechanistic framework to understand host filtering. Some of the clearest examples of these mechanisms come from the field of plant pathology, where plant traits can be used to predict disease outcomes (Fahey et al., 2020). One obvious suite of traits to study are plant defenses. As agents of plant regeneration, seeds are one of the most defended plant organs, protected by both chemical and physical defenses (Zangerl and Bazzaz, 1992; Fuerst et al., 2014; Fricke and Wright, 2016; Wang et al., 2018). Some of these defenses come from the mother plant, such as through innate floral defenses in angiosperms (Rhoades and Cates, 1976). Many studies on plant defense traits are obviously focused on protection against pests and pathogens (Dalling et al., 2020), but can be extended to other members of the microbial community (Fort et al., 2021).

A number of studies have been conducted to test how microbes interact with seeds at the micro-scale. Using microscopy, the microbial communities within seeds of *Citrullus lanatus* (Dutta et al., 2012) and *Q. petraea* (Fort et al., 2021) were found to differ in abundance and composition depending on seed sub-structure. Since *Q. petraea* is a wind-pollinated species, the variation in seed sub-structure colonization observed by Fort et al. (2021) suggests physical filtering of microbes during vertical and horizontal transmission. Although few studies have explored the role of micromorphology of developing seeds in microbial community acquisition (e.g., Eyre et al., 2019), there are plenty examples of such micro-scale studies come from work on the floral microbiome. Spinelli et al. (2005) used microscopy and fluorescent tagging to study the growth and movement of the bacteria *Erwinia amylovora* and *Pantoea agglomerans* on flowers of apple (*Malus domestica*) and pear (*Pyrus communis*). They found that the bacteria migrate from the stigma to the nectaries along a stylar groove in both species, indicating topographical effects on survival, population growth, and dispersal (Spinelli et al., 2005). Similarly, Steven et al. (2018) characterized at the high spatial resolution the floral bacterial communities on apple (*M. domestica*) using next-generation sequencing and found that different flower parts were enriched with different bacterial families (Steven et al., 2018). It is intriguing to think that variation in microtopography on flowers and stigmas may contribute to host filtering during the process of flower-to-seed horizontal transmission of microorganisms.

### Species interactions

The role of species interactions in metacommunity dynamics is important, but often overlooked in metacommunity ecology studies (Leibold et al., 2020). In plant microbiota research in general, much focus has been on pathogen antagonism interactions, for example with an eye toward applications in disease control (Busby et al., 2016). However, there is much interest and opportunity to better understand interactions between and among non-pathogens in plant and also seed microbial communities. As with traditional ecology studies, much of the work on species interactions in seed microbial communities focuses on competition and antagonism. For example, Raghavendra et al. (2013) inoculated *Centaurea stoebe* flowers with pairs of fungi and then cultured those fungi out of mature seeds. They always isolated the same single fungus from each pairing out of seeds across parent genotypes, and proposed that competition was the primary driver of selection (Raghavendra et al., 2013). Fungi compete for space and resources in *Q. petraea* seeds (Fort et al., 2021), and have negative interactions with bacteria in *Populus trichocarpa* seeds (Heitmann et al., 2021). Similar competitive exclusion has been observed in floral stigma communities (Cui et al., 2020), and in dormant seeds within the soil (Fuerst et al., 2018). However, seed microbes can also coexist *via* niche partitioning and other interactions. For example, Torres-Cortés et al. (2019) looked at how transmission of several bacterial pathogens impacted the composition of *Raphanus sativus* seed microbiomes. They found that these pathogens did not alter the composition of the seed microbiome, suggesting that differences in resource usage (niche partitioning) lead to coexistence between taxa (Torres-Cortés et al., 2019). A more complete understanding of the types and outcomes of microbial species interactions prior to and during seed development is desirable.

## Stochastic processes: Dispersal and ecological drift

### Dispersal

As with filtering, microbial dispersal to seeds occurs at multiple nested spatial scales, with different mechanisms at play for each spatial scale. For example, at the micro-scale, dispersal from floral stigmas to seeds can be impacted by variation in the level of protection or nutrients that are available to microbial colonizers, which is closely tied to stigma surface topography. The presence of pollen may also be important, as it has been shown that germinating pollen can enhance the flower-to-seed transmission of pathogens (Walcott et al., 2003) and that some bacteria can even induce pollen germination (Christensen et al., 2021). While there are no studies looking at the connection between floral topography and seed microbial transmission, experiments with flowers (Spinelli et al., 2005) and leaves (Doan et al., 2020) have demonstrated that bacterial dispersal is influenced by plant surface topography and surface water distribution. Conducting similar micro-scale inoculation experiments like these in flower-to-seed systems will illuminate how microbes actually move.

Seeing that microbes can be florally transmitted to seed, we need to consider studies on the dispersal of floral microbes to understand seed microbial communities at the meso-and macro-scales. One major finding from floral studies is that microbes are dispersal limited at regional scales (Belisle et al., 2012). For example, Belisle et al., 2012 found that yeast frequency in nectar communities of *Mimulus aurantiacus* was correlated with flower proximity, and they inferred that dispersal limitation was controlled by pollinator behavior. In a study on the floral microbiome across wildflower species of California, Vannette et al. (2020) observed that fungi were more dispersal limited between individual flowers and plant species than bacteria. Another major finding has been that pollinators can vector microbes between flowers and influence microbial community patterns. For example, Vannette and Fukami (2017) explored the variable effects of dispersal limitation on beta diversity in the nectar microbiome. Using a pollinator exclusion experiment, they found that increased dispersal by pollinators raised beta diversity and hypothesized that this increase was due to the stochasticity of dispersal timing which strengthens priority effects (Vannette and Fukami, 2017). A pollinator exclusion experiment in *B. napus* demonstrated that pollinators can also vector bacteria to seeds through flowers, impacting the local and regional diversity (Prado et al., 2020). These experiments indicate that dispersal may have unique effects on diversity in flower microbiome metacommunities *via* arrival history. However, all of these studies were performed only on the macro-scale, and they did not characterize dispersal traits. Furthermore, the association between dispersal patterns in floral microbial communities and those in seed communities has yet to be studied. Future experiments should explore if dispersal traits and arrival history consistently enhance beta diversity in flower and seed microbial communities among spatial scales (Vannette and Fukami, 2017).

### Ecological drift

Ecological drift is defined as random fluctuations in species abundances over time, and can be driven by random birth, death, and migration events (Leibold and Chase, 2018). Drift is particularly important when local communities are small (Fukami, 2004) and filtering is weak (Chase, 2010). This is key to note for seed microbial communities because they typically have low population sizes and low species richness (Newcombe et al., 2018; Bergmann and Busby, 2021). Random migration events may be particularly important for seed microbes, such as those vectored by rain or wind (Shade et al., 2017). However, while there is a lot of interest in drift and stochasticity in seed microbe research (Shade et al., 2017), drift as a process is difficult to study because it is hard to manipulate. Metacommunity ecologists have also generally found it difficult to get direct evidence of drift, with limited examples from experiments testing the coexistence of ecologically equivalent taxa (Leibold and Chase, 2018). One alternative approach to direct observation used in plant microbiota studies is to fit community data to neutral models, where community members are assumed to be ecologically equivalent, and non-significant variation in community composition across samples is explained by neutral processes. Rezki et al. (2018) took this approach when studying the seed microbiota of *R. sativus* by fitting fungal and bacterial community data to a Sloan neutral model (Sloan et al., 2007). This model accounts for neutral birth, death, and immigration rates, and estimates immigration rates into communities based on species frequencies across samples (Sloan et al., 2007). Immigration rates within the confidence interval of the predicted values imply that drift is structuring the community (Sloan et al., 2007). Based on the model, they found that bacterial community assembly was driven primarily by drift, while fungal communities were driven more by dispersal (Rezki et al., 2018). This study indicated that drift is important for some seed microbes, and more model-fitting studies or coexistence experiments are needed.

## Interactions between assembly processes across scales

At its core, metacommunity ecology emphasizes not only how the processes described above play out individually, but also how they interact with each other to produce emergent community patterns across scales. In plant microbiome research, the interaction between abiotic and host filters, also known as genotype-by-environment (GxE) interactions, has been of growing interest because it provides a more holistic explanation for microbiome variation (Wagner et al., 2016). Such an explanation can be applied to seed microbial communities, which may vary with seed nutrient profiles, osmotic stress, and water availability. However, as previously mentioned, GxE studies on plant microbiota face a scale problem where genotype and environment become synonymous at the micro-scale. Taking a plant trait-based approach to these studies may make the role of these effects more clear, and can connect micro-and macro-scales *via* host local adaptation.

While not emphasized as much as GxE interactions, the interaction between dispersal and filtering is also important during seed microbiome assembly. At the micro-scale, variation in the plant surface landscape (e.g., water availability and topography of the surface) can create differences in dispersal limitation between taxa. Doan et al. (2020) demonstrated this interaction on synthetic leaf surfaces, finding that surface water acted as a conduit for bacterial dispersal. This effect may also be present in floral stigmas, which are highly heterogeneous landscapes (Spinelli et al., 2005). Indeed, in their work on transmission of the pathogen *A. citrulli* from watermelon flowers and fruit to seeds, Dutta et al. (2012) found that inoculum from the flower dispersed more frequently and ended up in deeper seed tissues (e.g., endosperm, embryo) than inoculum from the fruit. While these examples suggest that heterogeneity in the plant landscape impacts dispersal limitation to seeds, more studies are needed.

Dispersal also intersects with species interactions, most clearly through historical contingency or priority effects (Fukami, 2015). In this phenomenon, the arrival order of community members dictates assembly outcomes, typically with an advantage to taxa that arrive first (Fukami, 2015). Priority effects can occur either through niche preemption, where the first colonizers fill all available niches, or by niche modification, where the first colonizers alter the environment and its resulting niches (Fukami, 2015). These effects are often cited as important in seed communities because they have few members (Bergmann and Busby, 2021). However, priority effect experiments in plant microbiota have typically been done in leaf (Leopold and Busby, 2020) and wood (Hiscox et al., 2015; Leopold et al., 2017) communities (Maignien et al., 2014). As such, there is a need to understand the role of priority effects in seed communities.

An exciting new approach for studying the multiple, interactive processes of dispersal, filtering, drift, and species interaction is with Joint Species Distribution Models (JSDMs), which extend single-species distributions to community-level dynamics (Ovaskainen et al., 2017). Leibold et al. (2020) used these models in tandem with variation partitioning to explain the internal structure of simulated metacommunities. They found that this approach was a promising way to connect metacommunity pattern data to multiple assembly processes (Leibold et al., 2020). In the seed microbiology literature, Fort et al. (2021) used JSDMs to infer how maternal filtering and abiotic filtering contributed to seed mycobiome (fungal microbiome) variation in *Q. petraea* seeds (i.e., acorns). They found that fungal guild (e.g., pathogen, saprotroph, etc.) influenced which taxa varied with abiotic filters, with elevation selecting saprotrophs and seed specialists, and all taxon co-occurrences were positive associations (Fort et al., 2021). While JSDMs were not used in a metacommunity context for this study, and they are limited in their omission of abundance data, these models provide an integrative approach for looking at seed microbiome assembly.

## Future directions

Multiple tools exist for exploring and exposing the effects and interactions of filtering, species interactions, dispersal, and drift on microbial community assembly of individual seeds at multiple spatial scales. However, future work can do a better job of integrating and connecting metacommunity ecology models to traditional seed microbial ecology studies at micro-, meso-and macro-scales. One technical challenge of taking this approach pertains to the interrogation of microbial communities in individual seeds. Culture-based studies of individual seeds report low isolation frequencies, with most seeds containing zero or one microbial taxon (Newcombe et al., 2018, 2022). Additionally, most sequence-based studies to date pool seeds by fields or other groupings (Rezki et al., 2018; Kim et al., 2020; Mascot-Gómez et al., 2021; Morales Moreira et al., 2021; Wassermann et al., 2022). As exceptions, Bergmann and Busby (2021) and Fort et al. (2021) sequenced fungi from individual tree seeds and found that sequencing depth was fairly high. However, the tree species in these studies produce large seeds; sequence-based detection of microbiota might be more difficult in small-seeded species (e.g., *Arabidopsis*). Additionally, it is often difficult or impossible to treat individual seeds as independent since experimental treatments or predictors are often applied at the fruit or plant level. To resolve these issues, future work could focus on species where seeds are fertilized independently (*Helianthus annuus, Quercus* sp.), or one seed per fruit/plant could be sampled for large-seeded species. Alternatively, seeds could be pooled at the fruit or plant levels, since these are the levels where treatments are often applied and they sufficiently capture the variation in seed microbiota (Bintarti et al., 2022) while still allowing for a metacommunity approach at the meso-and macro-scales. The appropriate level of pooling should be selected based on the transmission pathway of interest (i.e., fruit level for floral transmission, plant level for vertical transmission; Bintarti et al., 2022). Finally, seeds of large-fruited species could be pooled by parts of the fruit/pod for spatially explicit sampling at the meso-and micro-scales. These scale-explicit pooling approaches, along with the use of additional methods at the meso-and micro-scales (e.g., microscopy of individual seeds, inoculation experiments with synthetic communities), will allow for characterization of microbiota at or near the individual seed level while mitigating issues of low DNA amounts and cross-contamination.

At the micro-scale, there are many opportunities to take a traits-based approach to host filtering of seed microbiota. Experiments can go beyond studying if plant traits have an effect to testing what these effects are (e.g., changes in microbial colonization rates, fitness, dispersal, and species interactions). These experiments could also take a microbial trait-based approach to host filtering (and other processes) and identify the genes required for successful transmission, which are still largely unknown (Chesneau et al., 2020). This could provide valuable insights into the genes required for transmission across the different pathways (Chesneau et al., 2020). Furthermore, metagenomic analyses across plants, populations, species, etc., could determine if these transmission-associated genes are common across metacommunities. Such information could show if there is functional conservation across microbial communities, even if they are taxonomically variable. Finally, micro-scale experiments can also test how microbial community assembly is impacted by the interplay between deterministic and stochastic processes.

In addition to these tests of microbial and plant trait impacts, experiments testing the role of dispersal in seed microbial community assembly among spatial scales should be conducted. At the micro-scale, experiments using synthetic microbial communities on stigmas with varying chemistry and topography can demonstrate how dispersal and selection occur between flowers and seeds, and what the role is of plant genetics and microbial adaptations. At the macro-scale, pollinator exclusion experiments similar to those in Prado et al. (2020) could be conducted across sites in natural landscapes. By using sites at varying distances and connectivity levels from each other, and analyzing both within- and among-site seed microbial community variation, one may obtain new information about how pollinators and patch connectivity impact multi-scale dispersal ability. These proposed studies would elucidate how dispersal contributes to metacommunity assembly among spatial scales.

Along with these single-process studies, we envision studying the interactions between processes through both observational and experimental studies. As JSDMs continue to be refined to model nested and continuous metacommunities, they will provide a way to analyze seed microbiome patterns and their associated assembly processes that is more sophisticated than previous modeling approaches. Additionally, priority effect experiments conducted at multiple points in the seed life cycle (e.g., pollination, dispersal, germination) may reveal how historical contingencies impact seed microbiome assembly throughout the seed life cycle. Such experiments would also test the Primary Symbiont Hypothesis (Newcombe et al., 2018), which argues that seed communities are dominated by a single microbe with significant functional consequences for the plant.

Finally, questions will need to be asked about seed microbiome assembly that go beyond just testing for spatial mechanisms. Primary among these questions is: what fitness benefit does transmission into seeds provide to microbes and their host plants? Such a question gets at the eco-evolutionary dynamics in these microbial metacommunities, which can have long-term consequences for both microbes and plants. Because microbial communities behave and evolve at shorter time-scales than macro-organisms (Nemergut et al., 2013), it is feasible to design simple experiments testing how microbes evolve in response to plant defenses, nutrient availability, and micromorphology. Such eco-evolutionary studies may have applications in understanding microbial community shifts with crop domestication (Kim et al., 2020). Additionally, both microbes and seeds have dormant stages, which can impact metacommunity dynamics through tradeoffs with dispersal and delayed responses to environmental conditions (Wisnoski et al., 2019). The role of dormancy in seed and plant microbial metacommunity assembly has yet to be explored, so there is much room to study how dormancy impacts these systems over longer temporal scales. Finally, a hot topic in plant microbiome research is how to modify plant microbial communities for climate resilience and other beneficial traits (Busby et al., 2017; Mitter et al., 2017). However, the impacts of climate change-associated disturbances on plant microbiomes have been limited to pattern-based studies in leaves and roots (e.g., Kivlin et al., 2013). As such, more work can be done on how disturbances alter seed microbiome assembly processes and outcomes.

## Conclusion

Seed microbial community assembly, much like the assembly of microbial communities associated with other plant parts, is the result of complex interactions between multi-scale processes. Metacommunity ecology provides a conceptual framework for identifying these processes, and provides new statistical and theoretical tools for testing their interactions. We advocate for the spatially explicit, multi-scale study of seed microbial community assembly, with emphasis on the effects of plant topography and chemistry on micro-scale dispersal and persistence, the role of pollinators and seed dispersers on macro-scale dispersal, and the interactions between processes. These new avenues of study will provide a more generalizable understanding of seed microbiome assembly, with potential applications in plant conservation and sustainable agriculture.

## Author contributions

GB wrote and revised the manuscript and created the manuscript figure and tables. JL revised the manuscript and associated tables. All authors contributed to the article and approved the submitted version.

## Conflict of interest

The authors declare that the research was conducted in the absence of any commercial or financial relationships that could be construed as a potential conflict of interest.

The handling editor KM declared a shared affiliation with the authors at the time of review.

## Publisher’s note

All claims expressed in this article are solely those of the authors and do not necessarily represent those of their affiliated organizations, or those of the publisher, the editors and the reviewers. Any product that may be evaluated in this article, or claim that may be made by its manufacturer, is not guaranteed or endorsed by the publisher.
