# Supplementary material for: A metacommunity ecology approach to understanding microbial community assembly in developing plant seeds
Source: Front Microbiol. 2022 Jul 22;13:877519. doi: 10.3389/fmicb.2022.877519 (PMC9355165; doi:10.3389/fmicb.2022.877519)
Supplement: Supplementary file 1 [file Table_1.DOCX]

Supplemental Table 1. Common seed fungal and bacterial taxa reported across papers surveying the seed microbiota of various plant species.

| **Reference** | **Host plant** | **Plant group** | **Common fungi** | **Common bacteria** | **Survey method** |
| --- | --- | --- | --- | --- | --- |
| Bergmann, G.E. and Busby, P.E., 2021. The core seed mycobiome of Pseudotsuga menziesii var. menziesii across provenances of the Pacific Northwest, USA. *Mycologia*, *113*(6), pp.1169-1180. | Pseudotsuga menziesii | gymnosperm | *Hormonema, Trichoderma* | *NA* | culture and sequence-based |
| Bintarti, A.F., Kearns, P.J., Sulesky, A. and Shade, A., 2020. Abiotic treatment to common bean plants results in an altered seed microbiome. *bioRxiv*. | Phaseolus vulgaris | dicot | *Helvella, Gautieria* | *Pseudomonas, Bacillus* | sequence-based |
| Chartrel, V., Dugat-Bony, E., Sarthou, A.S., Huchette, S., Bonnarme, P. and Irlinger, F., 2021. The microbial community associated with pea seeds (*Pisum sativum*) of different geographical origins. *Plant and Soil*, *462*(1), pp.405-427. | Pisum sativum | dicot | *Cladosporium, Alternaria, Stemphylium* | *Pantoea, Pseudomonas, Erwinia* | culture and sequence-based |
| Fort, T., Pauvert, C., Zanne, A.E., Ovaskainen, O., Caignard, T., Barret, M., Compant, S., Hampe, A., Delzon, S. and Vacher, C., 2021. Maternal effects shape the seed mycobiome in Quercus petraea. *New Phytologist*, *230*(4), pp.1594-1608. | *Quercus patraea* | dicot | *Gnomoniopsis* | *NA* | sequence-based |
| Guo, J., Ling, N., Li, Y., Li, K., Ning, H., Shen, Q., Guo, S. and Vandenkoornhuyse, P., 2021. Seed‐borne, endospheric and rhizospheric core microbiota as predictors of plant functional traits across rice cultivars are dominated by deterministic processes. *New Phytologist*, *230*(5), pp.2047-2060. | Oryza sativa | monocot | *NA* | *Herbaspirillum, Acidovorax, Stenotrophomonas, Pseudoxanthomonas, Pantoea, Pseudomonas, Rhizobium, Sphingomonas, Microbacterium, Paenibacillus* | sequence-based |
| Heitmann, S., Bergmann, G.E., Barge, E., Ridout, M., Newcombe, G. and Busby, P.E., 2021. Culturable seed microbiota of *Populus trichocarpa*. *Pathogens*, *10*(6), p.653. | Populus trichocarpa | dicot | *Diaporthe, Cladosporium, Aureobasidium* | *Pseudomonas* | culture-based |
| Hone, H., Mann, R., Yang, G., Kaur, J., Tannenbaum, I., Li, T., Spangenberg, G. and Sawbridge, T., 2021. Profiling, isolation and characterisation of beneficial microbes from the seed microbiomes of drought tolerant wheat. *Scientific reports*, *11*(1), pp.1-12. | *Triticum sp.* | monocot | *NA* | *Pantoea, Pseudomonas, Arthrobacter, Curtobacterium* | culture and sequence-based |
| Morales Moreira, Z.P., Helgason, B.L. and Germida, J.J., 2021. Environment has a stronger effect than host plant genotype in shaping spring *Brassica napus* seed microbiomes. *Phytobiomes Journal, 5(2),* pp. 220-230. | *Brassica napus* | dicot | *Filobasidium, Alternaria, Mycosphaerella, Chalastospora, Vishniacozyma, Dioszegia, Stemphylium* | *Sphingomonadales, Enterobacteriales, Pseudomonadales,* | sequence-based |
| Chesneau, G., Torres-Cortes, G., Briand, M., Darrasse, A., Preveaux, A., Marais, C., Jacques, M.A., Shade, A. and Barret, M., 2020. Temporal dynamics of bacterial communities during seed development and maturation. *FEMS microbiology ecology*, *96*(12), p.fiaa190. | *Phaseolus vulgaris, Raphanus sativus* | dicot | *NA* | *Pseudomonas* | sequence-based |
| Guo, J., Bowatte, S. and Hou, F., 2021. Diversity of endophytic bacteria and fungi in seeds of Elymus nutans growing in four locations of Qinghai Tibet Plateau, China. *Plant and Soil*, *459*(1), pp.49-63. | *Elymus nutans* | monocot | *Epichloë, Pyrenophora* | *Pseudomonas* | sequence-based |
| Huet, S., Pouvreau, J.B., Delage, E., Delgrange, S., Marais, C., Bahut, M., Delavault, P., Simier, P. and Poulin, L., 2020. Populations of the parasitic plant Phelipanche ramosa influence their seed microbiota. *Frontiers in plant science*, p.1075. | *Phelipanche ramosa* | dicot | *Cladosporium* | *Pseudomonas, Stenotrophomonas, Pedobacter* | sequence-based |
| Prado, A., Marolleau, B., Vaissière, B.E., Barret, M. and Torres-Cortes, G., 2020. Insect pollination: an ecological process involved in the assembly of the seed microbiota. *Scientific reports*, *10*(1), pp.1-11. | *Brassica napus* | dicot | *NA* | *Acinetobacter, Pantoea, Sphingobium, Pseudomonas, Lactobacillus, Gillamella* | sequence-based |
| Rodríguez, C.E., Antonielli, L., Mitter, B., Trognitz, F. and Sessitsch, A., 2020. Heritability and functional importance of the Setaria viridis bacterial seed microbiome. *Phytobiomes Journal*, *4*(1), pp.40-52. | Setaria viridis | monocot | *NA* | *Burkholderia, Ralstonia* | sequence-based |
| Liu, Y., Yan, H., Zhang, X., Zhang, R., Li, M., Xu, T., Yang, F., Zheng, H. and Zhao, J., 2020. Investigating the endophytic bacterial diversity and community structures in seeds of genetically related maize (Zea mays L.) genotypes. *3 Biotech*, *10*(1), pp.1-10. | *Zea mays* | monocot | *NA* | *Enterobacter, Shigella, Pseudomonas, Achromobacter* | sequence-based |
| Deckert, R.J., Gehring, C.A. and Patterson, A., 2019. Pine seeds carry symbionts: endophyte transmission re-examined. In *Seed Endophytes* (pp. 335-361). Springer, Cham. | *Pinus edulis, Pinus ponderosa* | gymnosperm | *Clypeosphaeria, Rhizosphaera, Alternaria, Gymnopilus, Lactarius, Malassezia, Rhodotorula, Russula, Tomentella, Tubaria* | *Bacillus, Staphylococcus, Enterobacter, Erwinia, Pantoea, Sphingomonas, Pseudomonas, Lysinibacillus, Psychrobacillus* | culture-based |
| Eyre, A.W., Wang, M., Oh, Y. and Dean, R.A., 2019. Identification and characterization of the core rice seed microbiome. *Phytobiomes Journal*, *3*(2), pp.148-157. | *Oryza sativa* | monocot | *Alternaria, Hannaella* | *Methylobacterium, Sphingomonas* | sequence-based |
| Girsowicz, R., Moroenyane, I. and Steinberger, Y., 2019. Bacterial seed endophyte community of annual plants modulated by plant photosynthetic pathways. *Microbiological research*, *223*, pp.58-62. | *Carrichtera annua, Malva aegyptia, Trigonella stellate, Triticum aestivum, Stipa capensis, Suaeda aegyptium, Panicum miliaceum, Setaria verticillata, Setaria viridis, Opophytum forssakalii, Mesembbryanthemum nodiflorum, Aizoon canariense, Aizoon hispanicum, Sedum caespitosum* | monocot and dicot | *NA* | *Bacillus, Exiguobacterium, Terribacillus, Pomorum, Planococcus, Planomicrobium* | sequence-based |
| Vujanovic, V., Islam, M.N. and Daida, P., 2019. Transgenerational role of seed mycobiome–an endosymbiotic fungal composition as a prerequisite to stress resilience and adaptive phenotypes in Triticum. *Scientific reports*, *9*(1), pp.1-13. | *Triticum sp.* | monocot | *Aspergillus, Alternaria, Colletorichum, Fusarium, Stagonospora* | *NA* | sequence-based |
| Wassermann, B., Cernava, T., Müller, H., Berg, C. and Berg, G., 2019. Seeds of native alpine plants host unique microbial communities embedded in cross-kingdom networks. *Microbiome*, *7*(1), pp.1-12. | *Astrantia major, Euphrasia rostkoviana, Gentiana asclepiadea, Gentianella germanica, Heliosperma quadrifida, Parnassia palustris, Rhinanthus glacialis, Scabiosa lucida* | dicot | *Cladosporium, Cryptococcus, Davidiella* | *Tatumella, Pseudomonas* | sequence-based |
| Adam, E., Bernhart, M., Müller, H., Winkler, J. and Berg, G., 2018. The Cucurbita pepo seed microbiome: genotype-specific composition and implications for breeding. *Plant and soil*, *422*(1), pp.35-49. | *Cucurbita pepo* | dicot | *NA* | *Enterobacteriaceae* | sequence-based |
| Alibrandi, P., Cardinale, M., Rahman, M.D., Strati, F., Ciná, P., de Viana, M.L., Giamminola, E.M., Gallo, G., Schnell, S., De Filippo, C. and Ciaccio, M., 2018. The seed endosphere of Anadenanthera colubrina is inhabited by a complex microbiota, including Methylobacteriumspp. and Staphylococcus spp. with potential plant-growth promoting activities. *Plant and soil*, *422*(1), pp.81-99. | *Anadenanthera colubrina* | dicot | *NA* | *Methylobacterium, Staphylococcus* | culture and sequence-based |
| Bergna, A., Cernava, T., Rändler, M., Grosch, R., Zachow, C. and Berg, G., 2018. Tomato seeds preferably transmit plant beneficial endophytes. *Phytobiomes Journal*, *2*(4), pp.183-193. | *Solanum lycopersicum* | dicot | *NA* | *Burkholderiaceae* | sequence-based |
| Chen, H., Wu, H., Yan, B., Zhao, H., Liu, F., Zhang, H., Sheng, Q., Miao, F. and Liang, Z., 2018. Core microbiome of medicinal plant *Salvia miltiorrhiza* seed: a rich reservoir of beneficial microbes for secondary metabolism?. *International Journal of Molecular Sciences*, *19*(3), p.672. | *Salvia miltiorrhiza* | dicot | *Alternaria* | *Pantoea, Pseudomonas, Sphingomonas* | sequence-based |
| Rahman, M.M., Flory, E., Koyro, H.W., Abideen, Z., Schikora, A., Suarez, C., Schnell, S. and Cardinale, M., 2018. Consistent associations with beneficial bacteria in the seed endosphere of barley (Hordeum vulgare L.). *Systematic and applied microbiology*, *41*(4), pp.386-398. | *Hordeum vulgare* | monocot | *NA* | *Pantoea, Pseudomonas, Xanthomonas* | culture-based |
| Rezki, S., Campion, C., Simoneau, P., Jacques, M.A., Shade, A. and Barret, M., 2018. Assembly of seed-associated microbial communities within and across successive plant generations. *Plant and Soil*, *422*(1), pp.67-79. | *Raphanus sativus* | dicot | *Alternaria, Cladosporium* | *Pantoea, Pseudomonas* | sequence-based |
| Leff, J.W., Lynch, R.C., Kane, N.C. and Fierer, N., 2017. Plant domestication and the assembly of bacterial and fungal communities associated with strains of the common sunflower, *Helianthus annuus*. *New Phytologist*, *214*(1), pp.412-423. | *Helianthus annuus* | dicot | *Alternaria* | *Nocardiopsaceae,Enterobacteriaceae, Sphingomonadaceae* | sequence-based |
| Rybakova, D., Mancinelli, R., Wikström, M., Birch-Jensen, A.S., Postma, J., Ehlers, R.U., Goertz, S. and Berg, G., 2017. The structure of the *Brassica napus* seed microbiome is cultivar-dependent and affects the interactions of symbionts and pathogens. *Microbiome*, *5*(1), pp.1-16. | *Brassica napus* | dicot | *NA* | *Ralstonia* | sequence-based |
| Khalaf, E.M. and Raizada, M.N., 2016. Taxonomic and functional diversity of cultured seed associated microbes of the cucurbit family. *BMC microbiology*, *16*(1), pp.1-16. | *Curcurbita sp.* | dicot | *NA* | *Bacillus* | culture-based |
| Klaedtke, S., Jacques, M.A., Raggi, L., Préveaux, A., Bonneau, S., Negri, V., Chable, V. and Barret, M., 2016. Terroir is a key driver of seed‐associated microbial assemblages. *Environmental microbiology*, *18*(6), pp.1792-1804. | *Phaseolus vulgaris* | dicot | *Alternaria, Cladosporium, Fusarium, Cryptococcus, Eurotium* | *Pseudomonas* | sequence-based |
| Barret, M., Briand, M., Bonneau, S., Préveaux, A., Valière, S., Bouchez, O., Hunault, G., Simoneau, P. and Jacques, M.A., 2015. Emergence shapes the structure of the seed microbiota. *Applied and environmental microbiology*, *81*(4), pp.1257-1266. | *28 plants in Brassicaceae* | dicot | *Mycosphaerellaceae* | *Pantoea, Pseudomonas, Xanthomonas* | sequence-based |
| Brodal, G., Bye, H. R., Høst, E., and Talgø, V., 2015. Seed borne fungi on Christmas trees. In Proceedings from The 12th International Christmas Tree Research and Extension Conference (p. 38). | *Abies sp., Pinus sp.* | gymnosperm | *Hormonema, Sirococcus, Caloscypha* | *NA* | culture-based |
| Bentele, M., Morgenstern, K. and Krabel, D., 2014. *Lophodermium seditiosum* Minter, Staley & Millar seed-borne on Pinus sylvestris. *J. For. Landsc. Res*, *1*, pp.1-8. | *Pinus sylvestris* | gymnosperm | *Lophodermium seditiosum* | *NA* | sequence-based |
| Hodgson, S., de Cates, C., Hodgson, J., Morley, N.J., Sutton, B.C. and Gange, A.C., 2014. Vertical transmission of fungal endophytes is widespread in forbs. *Ecology and evolution*, *4*(8), pp.1199-1208. | *Centaurea cyanus, Centaurea nigra, Papaver rhoeas, Plantago lanceolata, Rumex acetosa, Senecio vulgaris* | dicot | *Cladosporium, Alternaria* | *NA* | culture-based |
| Links, M.G., Demeke, T., Gräfenhan, T., Hill, J.E., Hemmingsen, S.M. and Dumonceaux, T.J., 2014. Simultaneous profiling of seed‐associated bacteria and fungi reveals antagonistic interactions between microorganisms within a shared epiphytic microbiome on *Triticum* and *Brassica* seeds. *New Phytologist*, *202*(2), pp.542-553. | *Triticum sp., Brassica sp.* | monocot and dicot | *Alternaria* | *Pantoea, Masilia, Porphyrobacter, Pseudomonas, Pyrenophora, Sphingomonas, Telluria, Xanthomonas* | culture and sequence-based |
| Liu, Y., Zuo, S., Zou, Y., Wang, J. and Song, W., 2013. Investigation on diversity and population succession dynamics of endophytic bacteria from seeds of maize (*Zea mays* L., Nongda108) at different growth stages. *Annals of microbiology*, *63*(1), pp.71-79. | *Zea mays* | monocot | *NA* | *Undibacterium, Pantoea, Limnobacter, Burkholderia* | sequence-based |
| Liu, Y., Zuo, S., Xu, L., Zou, Y. and Song, W., 2012. Study on diversity of endophytic bacterial communities in seeds of hybrid maize and their parental lines. *Archives of microbiology*, *194*(12), pp.1001-1012. | *Zea mays* | monocot | *NA* | *Pantoea, Sphingomonas, Stenotrophomonas* | sequence-based |
| Compant, S., Mitter, B., Colli-Mull, J.G., Gangl, H. and Sessitsch, A., 2011. Endophytes of grapevine flowers, berries, and seeds: identification of cultivable bacteria, comparison with other plant parts, and visualization of niches of colonization. *Microbial ecology*, *62*(1), pp.188-197. | *Vitis sp.* | dicot | *NA* | *Bacillus, Paenibacillus* | culture-based |
| Johnston-Monje, D. and Raizada, M.N., 2011. Conservation and diversity of seed associated endophytes in Zea across boundaries of evolution, ethnography and ecology. *Plos one*, *6*(6), p.e20396. | *Zea sp.* | monocot | *NA* | *Enterobacter, Methylobacteria, Pantoea, Pseudomonas, Clostridium, Paenibacillus* | culture and sequence-based |
| Talgø, V., Brodal, G., Klemsdal, S.S. and Stensvand, A., 2010. Seed borne fungi on *Abies* spp. *Seed science and Technology*, *38*(2), pp.477-493. | *Abies sp.* | gymnosperm | *Penicillium, Trichoderma, Trichothecium, Cladosporium, Sydowia* | *NA* | culture-based |
| Cottyn, B., Debode, J., Regalado, E., Mew, T.W. and Swings, J., 2009. Phenotypic and genetic diversity of rice seed‐associated bacteria and their role in pathogenicity and biological control. *Journal of Applied Microbiology*, *107*(3), pp.885-897. | *Oryza sativa* | monocot | *NA* | Coryneform, Pantoea, Pseudomonas | culture-based |
| Kaga, H., Mano, H., Tanaka, F., Watanabe, A., Kaneko, S. and Morisaki, H., 2009. Rice seeds as sources of endophytic bacteria. *Microbes and environments*, pp.0904220080-0904220080. | *Oryza sativa* | monocot | *NA* | Bacillus, Caulobacter, Kocuria, Micrococcus | culture-based |
| Ganley, R.J. and Newcombe, G., 2006. Fungal endophytes in seeds and needles of *Pinus monticola*. *Mycological research*, *110*(3), pp.318-327. | *Pinus monticola* | gymnosperm | *Cladosporium* | *NA* | culture-based |
| Cankar, K., Kraigher, H., Ravnikar, M. and Rupnik, M., 2005. Bacterial endophytes from seeds of Norway spruce (*Picea abies* L. Karst). *FEMS microbiology letters*, *244*(2), pp.341-345. | *Picea abies* | gymnosperm | *NA* | *Pseudomonas, Rahnella* | *culture-based* |
